# Linking the evolution of catalytic properties and structural changes in copper–zinc nanocatalysts using *operando* EXAFS and neural-networks†

**DOI:** 10.1039/d0sc00382d

**Published:** 2020-03-11

**Authors:** Janis Timoshenko, Hyo Sang Jeon, Ilya Sinev, Felix T. Haase, Antonia Herzog, Beatriz Roldan Cuenya

**Affiliations:** Department of Interface Science, Fritz-Haber Institute of the Max-Planck Society 14195 Berlin Germany roldan@fhi-berlin.mpg.de

## Abstract

Understanding the evolution of unique structural motifs in bimetallic catalysts under reaction conditions, and linking them to the observed catalytic properties is necessary for the rational design of the next generation of catalytic materials. Extended X-ray absorption fine structure (EXAFS) spectroscopy is a premier experimental method to address this issue, providing the possibility to track the changes in the structure of working catalysts. Unfortunately, the intrinsic heterogeneity and enhanced disorder characteristic of catalytic materials experiencing structural transformations under reaction conditions, as well as the low signal-to-noise ratio that is common for *in situ* EXAFS spectra hinder the application of conventional data analysis approaches. Here we address this problem by employing machine learning methods (artificial neural networks) to establish the relationship between EXAFS features and structural motifs in metals as well as oxide materials. We apply this approach to time-dependent EXAFS spectra acquired from copper–zinc nanoparticles during the electrochemical reduction of CO_2_ to reveal the details of the composition-dependent structural evolution and brass alloy formation, and their correlation with the catalytic selectivity of these materials.

## Abstract 2

A neural network is used to reveal composition-dependent structural evolution under *operando* conditions in CuZn nanocatalysts for CO_2_ electroreduction.

## Graphical abstract

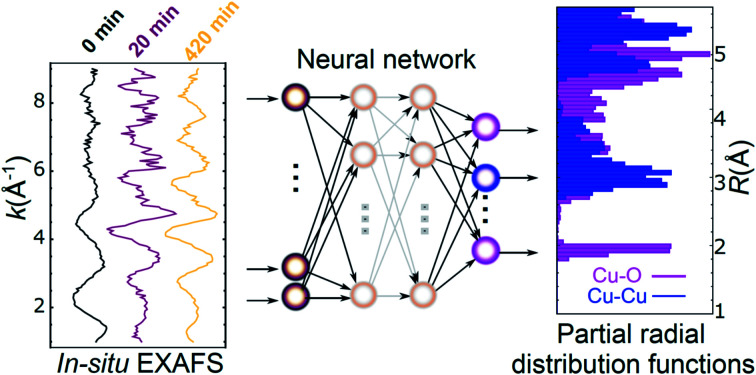

## 1. Introduction

Bimetallic nanoparticles (NPs) are attractive materials for applications in heterogeneous catalysis due to the high tunability of their catalytic properties. Coexistence of atoms of two (or more) types in a particle affects the catalytic properties due to the presence of multielement structural motifs and through the combination of geometric effects (*e.g.*, changes in lattice constants, surface strain, and enhanced structural disorder) and electronic (ligand) effects.^1–6^ In addition to the structural characteristics that are known to affect the catalytic activity and selectivity in monometallic systems (*e.g.*, oxidation state, structure, finite-size effects, particle–support interactions), the additional degrees of freedom in bimetallic materials provide new paths to control the interactions with adsorbates, resulting in unique catalytic properties and applications. For example, the addition of a secondary metal, such as zinc,^7–13^ is a common route to tailor the selectivity of copper and other metallic catalysts for the CO_2_ electroreduction reaction (CO_2_RR) and to steer the reaction towards the production of valuable chemicals and fuels.^14–23^

To understand the working mechanism of a bimetallic catalyst and the roles of various structural characteristics, the multielement structural motifs need to be detected and characterized experimentally. In such studies, *in situ* and *operando* investigations are essential, since the composition and structure of the catalyst can change significantly under working reaction conditions. X-ray absorption spectroscopy (XAS), and, in particular, extended X-ray absorption fine structure (EXAFS) analysis, is among the few experimental techniques that can be used to extract element-specific information about the oxidation state, composition and atomistic structure of catalytic species under reaction conditions.^24–29^ Interpretation of EXAFS data in such intrinsically disordered and heterogeneous catalyst materials is, however, non-trivial, and the conventional data analysis approaches that work well for simple, well-defined and well-ordered bulk materials may have limited accuracy and/or introduce artefacts, as pointed out by Clausen *et al.* and others.^30–39^

To address this problem, we have recently proposed a machine learning-based approach for the interpretation of experimental XAS data, where we employed artificial neural networks (NNs) to extract the structural information.^40–44^ In the case of EXAFS data interpretation, by exposing NN to thousands of training examples (theoretical EXAFS spectra, for which the corresponding structure was known) we could establish a relationship between spectral features and structure descriptors. The trained NN can then be used to directly invert experimental data, and quickly and accurately extract relevant structural information, such as partial radial distribution functions (RDFs). In our previous works, we demonstrated the power of this approach in studies of bulk metals,^45^ monometallic nanoparticles,^39,46^ as well as bimetallic model systems.^46^ However, the previous examples were limited to the applications of the NN-EXAFS method to completely metallic systems. The success of our method in these cases was ensured by the fact that we addressed relatively simple problems (the structure determination in close-packed metals). Applicability and practicality of this approach for oxidized and covalent systems, which exhibit larger varieties of possible structural motifs, or systems that are heterogeneous mixtures of reduced and non-reduced species, are, however, not guaranteed by these previous works. At the same time, investigations of such complex systems are clearly relevant for the understanding of real catalysts at work, and, especially, of catalysts transformations and their evolution under reaction conditions.

In this study we generalize the NN-EXAFS approach to oxidized and partially oxidized size-selected copper–zinc NP catalysts for applications in the CO_2_RR. We have recently shown that the catalytic selectivity of this system can be tuned by varying the copper to zinc ratio, and that significant structural changes take place under reaction conditions.^13,47^ Here we take advantage of the sensitivity of our NN-EXAFS method^44^ to monitor subtle chemical and structural changes taking place during the CO_2_RR as a function of the Cu–Zn NP composition and time. In particular, we will show that the reduction of copper and zinc species takes place on different time-scales, and that gradual alloying and a transition from a close-packed to a more disordered structure take place. Moreover, we will demonstrate that the NP composition affects not only the initial catalyst structure, but also its final state, transformation kinetics and consequently the catalyst stability and reactivity.

## 2. Experimental

CuZn NPs with an average size of *ca.* 5 nm and variable composition were synthesized by an inverse micelle encapsulation method. Synthesis and characterization details are given in ref. 13. The elemental composition was modified by controlling the molar ratio of the Cu and Zn precursor salts, CuCl_2_ and Zn(CH_3_COO)_2_. The NP composition was confirmed by XPS measurements under UHV conditions. Pre-formed CuZn NPs were deposited on glassy carbon substrates, and an O_2_-plasma treatment was used to remove the polymeric ligands. Average NP sizes (heights) were determined and the narrow size distributions were confirmed by atomic force microscopy (AFM, Bruker, Multimode 8).

CO_2_RR experiments were carried out in a purified, CO_2_-saturated 0.1 M KHCO_3_ electrolyte at −1.35 V *vs.* RHE in an H-type electrochemical cell separated by an anion-exchange membrane (Selemion AMV) with a Pt mesh counter electrode and a leak-free Ag/AgCl reference electrode. An Autolab potentiostat (Multi Autolab M204) was used. The gas products were quantified by gas chromatography (Agilent 7890B) equipped with thermal conductivity and flame ionization detectors. The formic acid concentration was analysed by high-performance liquid chromatography (Shimadzu Prominence) equipped with a NUCLEOGEL® SUGAR 810 column and a refractive index detector.

*In situ* XAS measurements were performed at the SAMBA beamline at SOLEIL synchrotron (for Cu_50_Zn_50_ and Cu_30_Zn_70_ NPs), and at CLAESS beamline at ALBA synchrotron (for Cu_100_, Zn_100_, and Cu_70_Zn_30_ NPs). A home-made electrochemical cell was used for the *operando* XAS measurements. The samples were measured at −1.35 V *vs.* RHE in a CO_2_ saturated 0.1 M KHCO_3_. XAS spectra for the Cu K-edge (*E*_0_ = 8979 eV) and Zn K-edge (*E*_0_ = 9659 eV) were collected separately. Identical but fresh samples were used for measurements at the Cu and Zn absorption edges. Time-resolved spectra were acquired with 8–12 minutes acquisition time per spectrum, until no further changes were observed in the data. The times required to achieve a “steady-state” structure/composition were different for samples with different Cu/Zn ratios. Alignment, background subtraction and normalization of the XAS spectra were performed using the conventional approach as implemented in the Athena software.^48^ Further details of the XAS measurements are given in the electronic ESI (Note 1†).

## 3. Results

### 3.1 Neural network-based analysis of EXAFS data

For the extraction of structural information from experimental EXAFS data we follow the general approach introduced and validated for metallic systems in our previous works.^40,44,46^ Using an artificial NN, we invert the EXAFS equation that links the measured spectrum *χ*(*k*) to the partial radial distribution functions (RDFs) *g*_*p*_(*R*):1
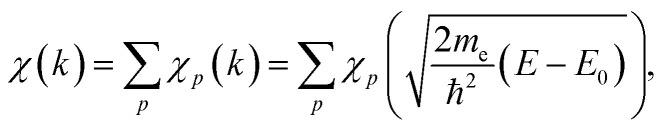
where2
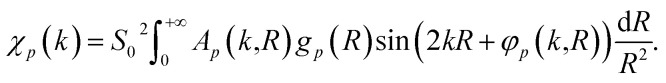


Here, *m*_e_ is the electron mass, *ħ* is the Planck's constant, *E* is the X-ray photon energy and *E*_0_ is the photoelectron reference energy. *A*_*p*_ and *φ*_*p*_ are the scattering amplitude and phase functions that can be calculated theoretically with codes like FEFF,^49^ and ensure chemical sensitivity of EXAFS. S_0_^2^ is the amplitude reduction factor due to many-electronic effects. Eqn (2) applies to single scattering contributions, but can be generalized to describe also the contributions of multiple scattering paths. In the latter case, *A*_*p*_ and *φ*_*p*_ depend not only on the interatomic distances *R*, but also on relative positions of the atoms (*e.g.*, bonding angles).^50^

The RDFs *g*_*p*_(*R*) contain key information about the local structure of the material – average number of atoms of a given type around the absorbing atom, information about the interatomic distances, crystallographic structure, oxidation state, and disorder. We distinguish between the partial contributions of, *e.g.*, Cu–O and Cu–Cu, because the scattering functions *A*_Cu–O_ (and *φ*_Cu–O_) are substantially different from *A*_Cu–Cu_ (and *φ*_Cu–Cu_). Consequently, the corresponding partial RDFs *g*_Cu–O_(*R*) and *g*_Cu–Cu_(*R*) contribute differently to the experimental spectrum and can be determined independently. The difference between the scattering functions of elements that are neighbours in the periodic table (*e.g.*, Cu and Zn) is, however, negligible, and we cannot distinguish between them. Therefore, for bimetallic systems we use here the total RDF *g*_Cu–M_(*R*) instead of *g*_Cu–Zn_(*R*) and *g*_Cu–Cu_(*R*) RDFs, where M is either Cu or Zn.

Using the NN method, we express the RDFs, parametrized as a histogram of bond-lengths with bin size Δ*R*, as *g̃*_*p*_(*R*_*i*_) = *F*_*i*_(*Θ*, *χ*(*k*)), where *F*_*i*_ is a non-linear composite function that can be represented as a network of nodes. The values of the nodes in the first NN layer (input layer) correspond to the values of *χ*(*k*) pre-processed *via* a wavelet transform^51^ (see the ESI Note 2, for details†). The values of the *i*-th node in the *n* + 1 NN layer can be obtained as: 
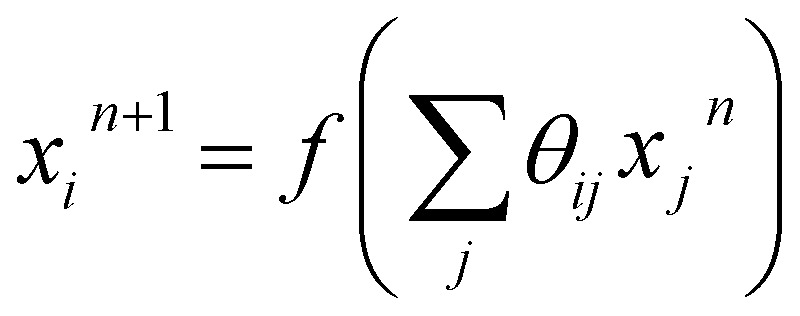
, where *f* is the so-called activation function. *Θ* is the set of all weights *θ*_*ij*_ that need to be found during the NN training step. The last NN layer (output layer) yields the heights of corresponding bins in the *g̃*_*p*_(*R*_*i*_) histogram.

To establish the values of the *θ*_*ij*_ weights we exposed the NN to a large set of training spectra, for which the true values of the corresponding *g*_*p*_(*R*_*i*_) are known, and we optimized *θ*_*ij*_*via* a back-propagation algorithm^52^ to achieve the best possible agreement between the true *g*_*p*_(*R*_*i*_) and the NN-yielded values *g̃*_*p*_(*R*_*i*_). The NN method thus relies on the availability of such a set of representative training spectra. For metallic materials, we have demonstrated that these spectra can be generated in classical molecular dynamics (MD) simulations.^40,44,46^ For close-packed metals, MD employing simple interaction models, such as the Sutton-Chen force (SC) field model,^53^ results in realistic material structure models, for which both, the RDFs and the corresponding time- and sample-averaged EXAFS spectra *χ*(*k*) can be easily calculated, allowing us to establish the relationship between EXAFS features and RDF, and, consequently, to extract RDFs from experimental EXAFS data. No generally applicable empirical force-field model, however, exists for oxides and other covalent materials, due to the significantly more complex and strongly directional nature of the chemical bond in these materials. In some specific cases, such as wurtzite-type ZnO and rocksalt-type NiO, an accurate description of the materials structure and dynamics can be achieved in MD with Buckingham-type potentials, and, consequently, theoretical EXAFS spectra can be generated that, as we have demonstrated,^54–59^ match well the corresponding experimental data.

**Fig. 1:**
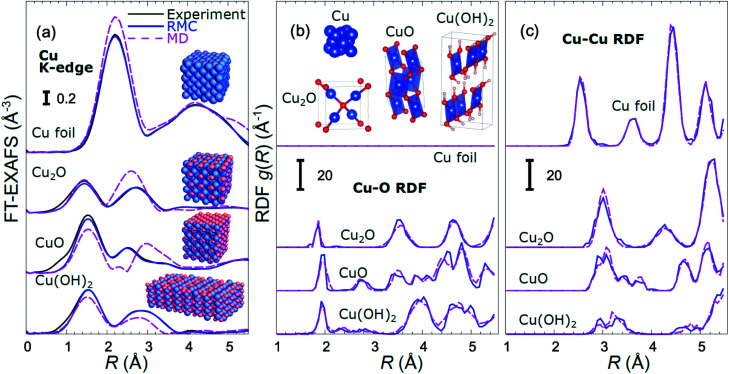
Comparison of Fourier-transformed (FT) experimental Cu K-edge EXAFS data for reference materials with the results of MD simulations and RMC fitting (a). Insets in (a) show the final structure models obtained in RMC calculations. RDFs yielded by MD and RMC methods are compared in (b) for Cu–O bonds, and in (c) for Cu–Cu bonds. The differences in bonding motifs in reference materials used here are shown in inset in (b).

For copper oxides, different empirical potential models have been proposed in the literature, starting from a simple Lennard-Jones (LJ) type potential,^60^ ending up with advanced charge optimized many body (COMB)^61^ and reaxFF-type potentials.^62^ We used MD calculations with a reaxFF potential to simulate the CuO and Cu(OH)_2_-type structures, the LJ-type potential for the Cu_2_O-type structures, and the SC-type potential for the metallic close-packed (fcc and hcp) structures. Examples of EXAFS spectra for bulk reference materials, calculated with these interaction models, together with the corresponding RDFs are shown in Fig. 1 and in the ESI, Fig. S1.† For completeness, we also included in our training dataset models with rocksalt-type structure (as in NiO), wurtzite-type structure (as in ZnO) and anti-perovskite type structure (as in Cu_3_N) using force-field models previously validated by us with experimental EXAFS data.^54–57,63^ The use of the materials with such a large variety of possible bonding motifs (inset in Fig. 1(b)) for NN training expands the applicability of our method. The details of the MD simulations, generation of theoretical EXAFS spectra and further discussion of the MD-EXAFS accuracy are described in ESI Note 3.†

An important step before we apply the NN for the interpretation of real experimental data of interest is to validate its accuracy by applying it to the analysis of experimental data of reference materials, for which the corresponding structure is known, or can be extracted by other methods. As in our previous works,^39,45,46^ for this purpose we rely on reverse Monte Carlo (RMC) simulations,^50,64–66^ which for bulk materials with well-defined structures provide an alternative way to fit experimental EXAFS data (Fig. 1(a)) and extract RDFs (Fig. 1(b and c)), and can thus be used for NN validation. Note that the RDFs extracted by the RMC method from experimental data for reference compounds are close to those obtained in MD simulations (Fig. 1(b and c)), confirming that the MD models provide a realistic description of metallic and oxidized copper compounds, and can be used for NN training. The details of the NN validation by the RMC method are given in ESI Note 4.† The good agreement between RMC and NN results for pure reference compounds (Fig. S2†) and their mixtures (Fig. S3†) gives us confidence in the accuracy of the proposed approach. Importantly, while our NN is trained on theoretical Cu K-edge EXAFS data in various Cu-based materials only, due to the similarity of the scattering functions of elements that are neighbours in the periodic table, it can be immediately used to extract total Cu–metal (Cu–M) RDFs in copper–zinc brass alloys, as well as Zn–O and Zn–M RDFs in oxidized and metallic Zn-based materials from the corresponding Zn K-edge EXAFS spectra (Fig. S2†). In the next sections, the data analysis approaches discussed here will be applied to the analysis of *operando* EXAFS data from pre-oxidized Cu–Zn NPs.

### 3.2 Structure–reactivity correlations in CO_2_RR

**Fig. 2:**
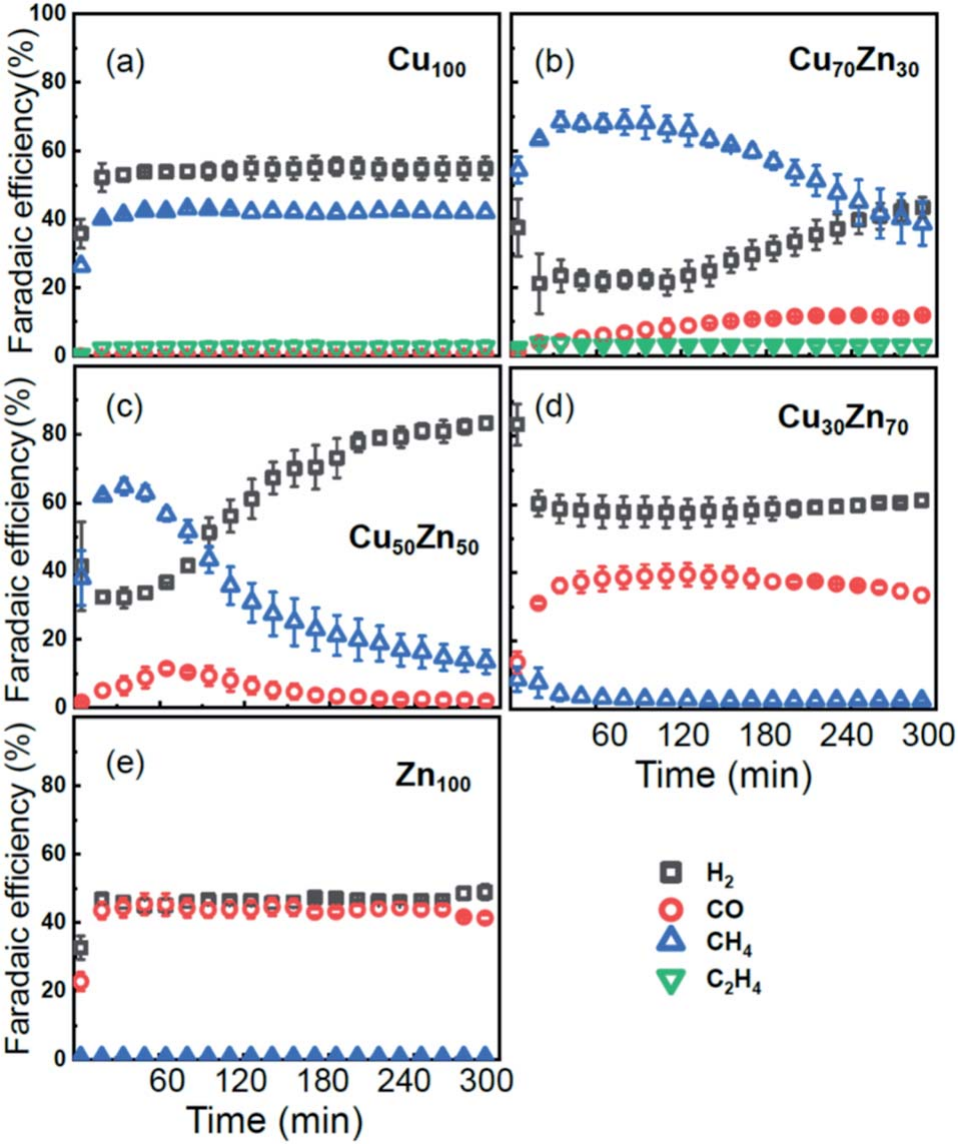
Time-dependencies of the Faradaic efficiency of the main reaction products for CO_2_RR at −1.35 V *vs.* RHE for Cu NPs (a), Cu_70_Zn_30_ NPs (b), Cu_50_Zn_50_ NPs (c), Cu_30_Zn_70_ NPs (d) and Zn NPs (e).

As discussed in our previous study,^13^ different catalytic selectivities for the CuZn NPs were obtained as a function of the Cu to Zn ratio. Moreover, the new experimental data in Fig. 2 demonstrate that the selectivity trends are time dependent and clearly different for samples with different composition. Pure Cu and Zn NPs display an overall stable production of CH_4_ and CO, respectively, while significant variations in the catalyst selectivity were observed within the first few reaction hours for the bimetallic samples.

**Fig. 3:**
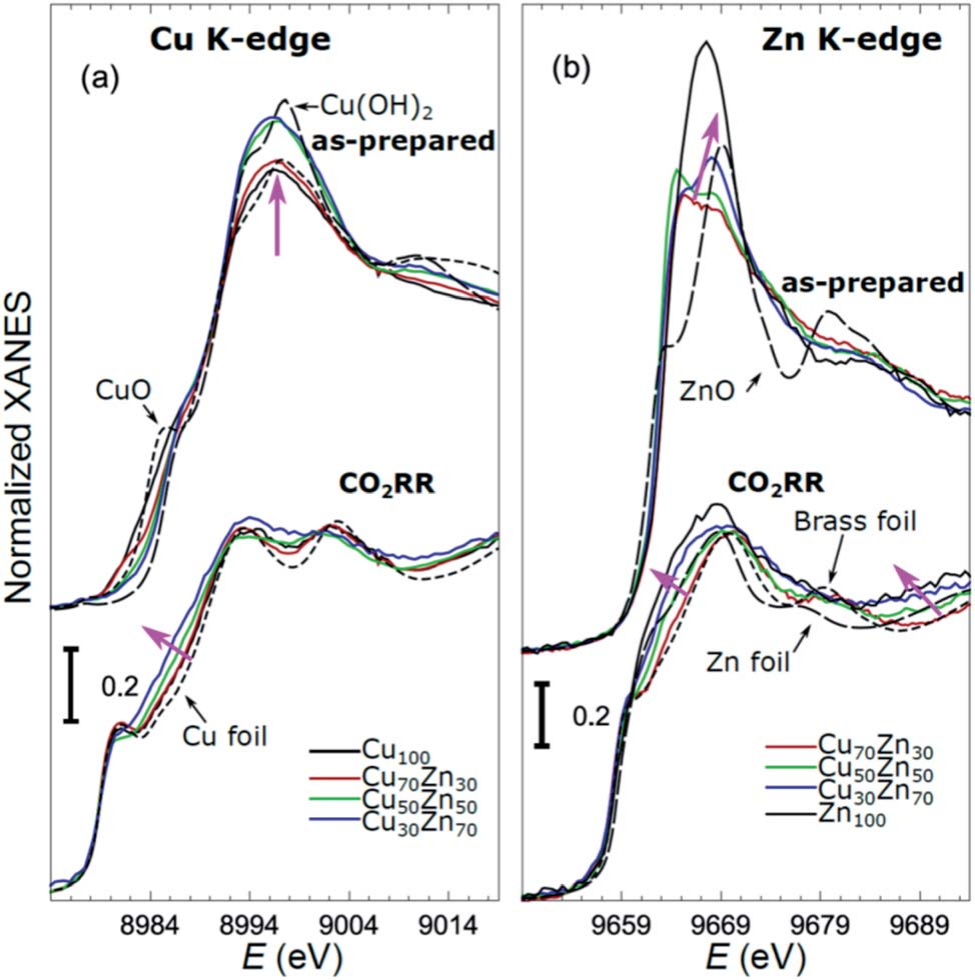
Cu K-edge (a) and Zn K-edge (b) XANES spectra of CuZn NPs with different composition. Spectra for as-prepared samples and for samples in their final state after 1–7 hours under CO_2_RR conditions are shown together with those for reference materials (ZnO, CuO, Cu(OH)_2_, Zn foil, Cu foil, CuZn brass foil (Cu to Zn ratio 7 : 3)). The spectra are shifted vertically for clarity. Magenta arrows emphasize the changes in the spectra with increasing Zn content.

**Fig. 4:**
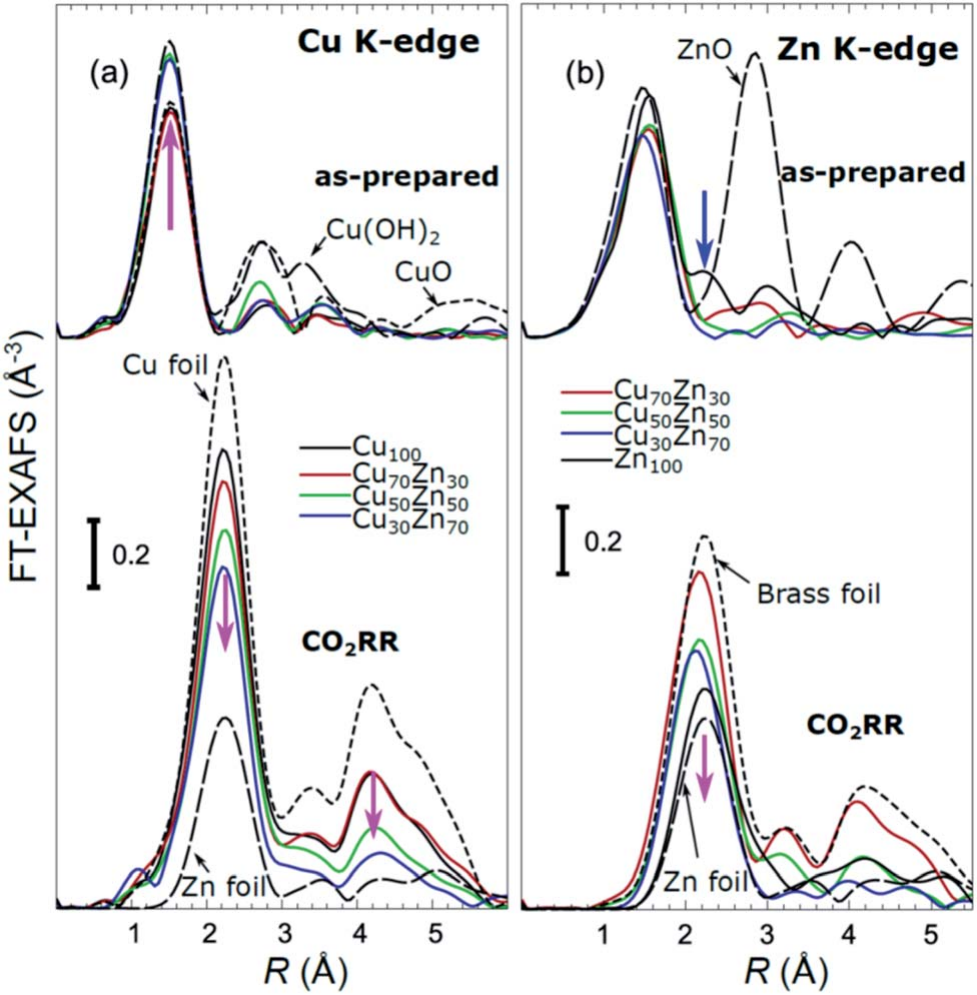
Cu K-edge (a) and Zn K-edge (b) Fourier-transformed (FT) EXAFS spectra or CuZn NPs with different composition. Spectra for as-prepared samples and for samples in final state after 1–7 hours under CO_2_RR conditions are shown together with those for reference materials (ZnO, CuO, Cu(OH)_2_, Zn foil, Cu foil, CuZn brass foil (Cu to Zn ratio 7 : 3)). The spectra are shifted vertically for clarity. Magenta arrows emphasize the changes in the spectra with increasing Zn content. Note that the spectra shown are “phase-uncorrected”, thus the positions of the FT-EXAFS peaks do not correspond to the true positions of the RDF peaks.

X-ray absorption near edge structure (XANES) spectra collected at the Cu K-edge and Zn K-edge for CuZn NPs with different composition are compared in Fig. 3. Spectra for as-prepared samples and for samples in their final state after several hours under CO_2_RR conditions are included. Corresponding Fourier-transformed (FT) EXAFS spectra for these samples are shown in Fig. 4. The time-dependency of the Cu K-edge and Zn K-edge EXAFS spectra is shown in Fig. S4.† Preliminary EXAFS data analysis for some of the samples based on conventional Cu- and Zn K-edge EXAFS data fitting was presented in ref. 13 and 47.

Once the accuracy of the NN method has been validated as shown in ESI Note 4,† we can apply it for the interpretation of real experimental data of CuZn nanocatalysts. Due to the relatively large sizes of the NPs in our samples (*ca.* 5 nm), the EXAFS spectra and RDFs are practically not affected by the NP size, and our NN, trained on theoretical EXAFS data for bulk compounds, is adequate for the description of this system. For the analysis of smaller NPs (with sizes below 3 nm), truncated structure models can be included in the NN training, as demonstrated in our previous works.^39,46^

**Fig. 5:**
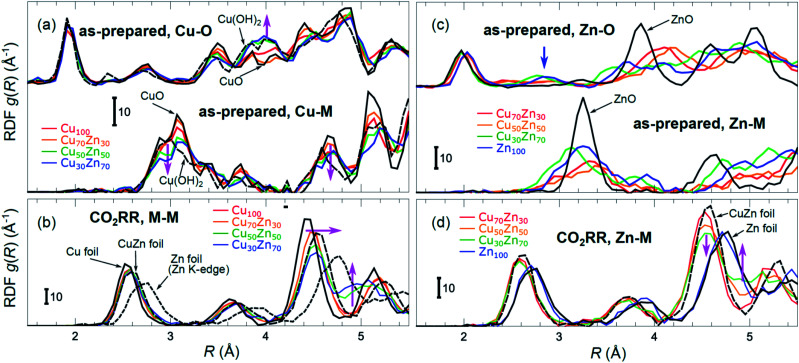
Cu–O and Cu–M (M = Cu or Zn) RDFs extracted by NN from experimental Cu K-edge EXAFS data (see Fig. 4(a) and S4(a)†) for Cu_100_, Cu_70_Zn_30_, Cu_50_Zn_50_ and Cu_30_Zn_70_ NPs in their as prepared state (a) as well as in their final state after 1–7 hours under CO_2_RR conditions (b). Zn–O and Zn–M RDFs extracted by NN from experimental Zn K-edge EXAFS data (see Fig. 4(b) and S4(b)†) for Cu_70_Zn_30_, Cu_50_Zn_50_, Cu_30_Zn_70_ and Zn_100_ NPs in their as-prepared state (c) as well as in their final state after 1–7 hours under CO_2_RR conditions (d). Data extracted from Cu K-edge EXAFS for reference compounds (Cu foil, CuZn brass foil, CuO and Cu(OH)_2_), as well as Zn–Zn RDF, extracted from Zn K-edge EXAFS for a Zn foil are also shown for comparison in (a) and (b). Zn–O and Zn–Zn RDFs extracted by the NN from experimental Zn K-edge EXAFS for reference compounds (Zn foil, CuZn brass foil and ZnO) are shown in (c) and (d). RDFs are shifted vertically for clarity.

**Fig. 6:**
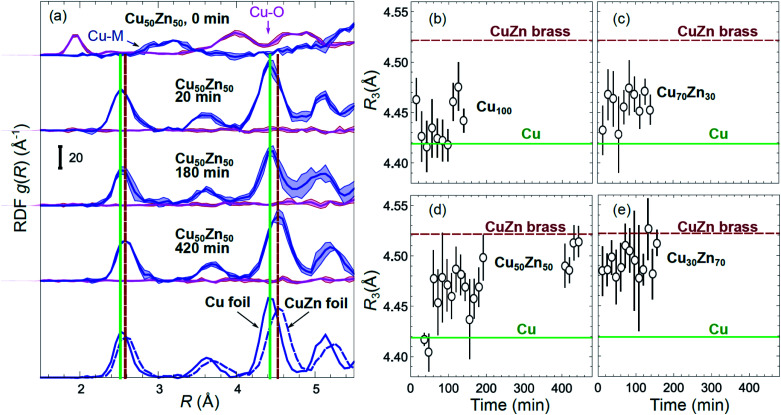
(a) Evolution of the partial RDFs for the Cu_50_Zn_50_ NP sample, as extracted by NN from time-dependent Cu K-edge EXAFS data (see Fig. 4(a) and S4(a)†). RDFs extracted from the Cu K-edge EXAFS of a Cu foil and a CuZn brass foil are shown for comparison. Vertical solid and dashed lines show the positions of the maxima for the 1^st^ and 3^rd^ RDF peaks in the Cu foil and CuZn brass foil, respectively. (b–e) The time dependencies of the positions of the 3^rd^ peak in the Cu–M RDF for monometallic Cu and bimetallic CuZn nanocatalysts.

Partial Cu–O and Cu–M (M = Cu, Zn) RDFs, extracted by NN from experimental Cu K-edge EXAFS data for as-prepared samples and for samples in their final state under CO_2_RR conditions (see Fig. 4(a)) are compared in Fig. 5(a and b). The time-dependent Cu K-edge EXAFS data for Cu_50_Zn_50_ NPs are analysed by the NN and the evolution of the RDFs obtained is shown in Fig. 6(a). Corresponding figures for other CuZn samples are shown in the ESI, Fig. S5.† A systematic shift of the RDFs features towards larger interatomic distances is observed under reaction conditions. This trend is illustrated in Fig. 6(b–e) and in the ESI, Table S1,† where the time-dependencies of the maximum point of the 3^rd^ Cu–M RDF peak (located at *ca.* 4.5 Å) for Cu_100_, Cu_70_Zn_30_, Cu_50_Zn_50_ and Cu_30_Zn_70_ NP samples are shown, corresponding to the interatomic distance in the 3^rd^ coordination shell in the metallic phase. Qualitatively similar corresponding time-dependencies of the position of the first RDF peak (peak at *ca.* 2.5 Å, corresponding to the first coordination shell) are shown in the ESI, Table S1 and Fig. S6.† We focus on the 1^st^ and 3^rd^ RDF peaks, because these are the most well-defined Cu–M RDF peaks in CuZn nanocatalysts that can also be singled out from contributions of other coordination shells.

**Fig. 7:**
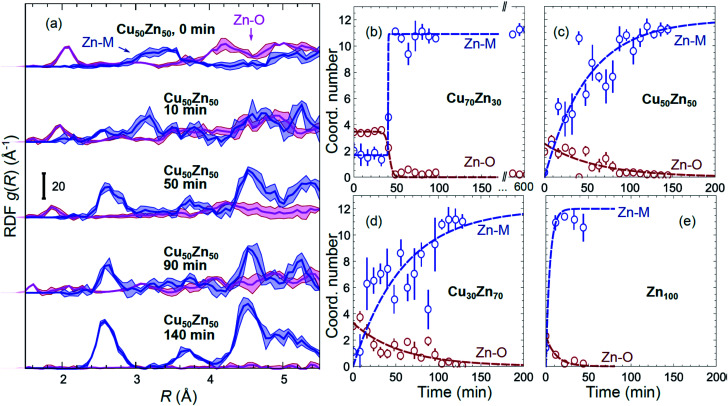
(a) Evolution of the partial RDFs for the Cu_50_Zn_50_ NPs as extracted by NN from time-dependent Zn K-edge EXAFS data (see Fig. 4(b) and S4(b)†). (b–e) The time-dependencies of the integrated areas under the 1^st^ Zn–O RDF peak (1^st^ shell Zn–O coordination number) and under the 1^st^ Zn–M RDF peak (1^st^ shell Zn–M coordination number) for CuZn nanocatalysts and for Zn_100_ NPs.

Partial Zn–O and Zn–M (M = Zn, Cu) RDFs, extracted by NN from experimental Zn K-edge EXAFS data for the NP samples in their as-prepared state as well as in their final state under CO_2_RR conditions (see Fig. 4(b)) are compared in Fig. 5(c and d). The time-dependent Zn K-edge EXAFS data for the Cu_50_Zn_50_ NPs are analyzed by a NN, and the evolution of the obtained RDFs is shown in Fig. 7(a). Corresponding figures for other CuZn samples are included in the ESI, Fig. S7.† The gradual decrease of the Zn–O contribution (first shell Zn–O coordination number), and increase of the Zn–M contribution (first shell Zn–M coordination number) under reaction conditions is shown in Fig. 7(b–e) and ESI, Table S2† for the Cu_70_Zn_30_, Cu_50_Zn_50_, Cu_30_Zn_70_ and Zn_100_ NP samples. Coordination numbers are obtained by integrating the RDFs, similarly as in Fig. S3.†

## 4. Discussion

While the selectivity of the monometallic Cu and Zn NPs does not change for 5 hours under CO_2_RR (Fig. 2(a and e)), for the Cu_70_Zn_30_ and Cu_50_Zn_50_ NPs we observe a decrease in the CH_4_ selectivity after a few hours, accompanied by, first, an increase of CO selectivity, and then H_2_ selectivity, Fig. 2(b and c). The change of the Cu_50_Zn_50_ NPs selectivity is more abrupt than that of the Cu_70_Zn_30_ NPs, with the production of CH_4_ for the former catalyst becoming suppressed completely after 5 hours under potential. For a sample with a lower Cu content, Cu_30_Zn_70_ NPs (Fig. 2(d)), a minor production of CH_4_ is observed only immediately after the potential is applied and is suppressed within minutes. No significant changes in catalyst selectivity for this sample are observed after that. The composition-dependency of the catalytic properties suggests the presence of different structural motifs in the investigated catalysts, while the pronounced time-dependencies of the selectivity highlight the role of transformations taking place in these materials under reaction conditions. To interpret these trends, we now turn to the discussion of the *operando* XAS data and their deciphering using a NN approach.

The main findings of our study are that differences in sample composition result in important differences in (i) the structure of as-prepared catalysts, (ii) the structure of the final state under CO_2_RR, as well as (iii) the rate and degree of structural and compositional changes under reaction conditions. In the as-prepared state, both, Cu and Zn are completely oxidized (+2 state, Fig. 3 and 4) in all samples, as evidenced by the similarity of the catalysts XANES and EXAFS data obtained with respect to those of CuO/Cu(OH)_2_ and ZnO. Nevertheless, when the data for catalysts with different Cu to Zn ratios are compared, we observe systematic differences, emphasized by the magenta arrows in Fig. 3 and 4. In particular, an increase in the amplitude of the Cu K-edge white-line and the amplitude of the main Cu K-edge FT-EXAFS peak for the Zn-rich Cu NPs was observed. The spectra at the Cu K-edge for the as-prepared Cu-rich and Zn-rich NPs are similar to the spectra for CuO and Cu(OH)_2_, respectively. The results obtained by our NN method confirm this observation. Note that due to the similarity of the 1^st^ coordination shell in CuO and Cu(OH)_2_ materials, it is difficult to distinguish between these materials using conventional EXAFS data analysis. However, it becomes possible when we employ the sensitivity of the NN to contributions of further coordination shells. As shown in Fig. 5(a), several regions in *R*-space can be identified, where Cu–O and Cu–Cu RDFs in CuO and Cu(OH)_2_ are substantially different. In particular, the Cu–O contribution is enhanced in Cu(OH)_2_ at *R* values of *ca.* 4 Å, while the Cu–Cu contribution is suppressed at *R* values *ca.* 3 and 4.5 Å. In all these regions we obtained for the CuZn nanocatalysts the systematic transformation from a CuO-like structure to a Cu(OH)_2_-like structure with increasing Zn concentration, as highlighted by magenta arrows in Fig. 5(a).

In the case of the Zn environment in the as-prepared samples, in Fig. 3(b) we observe systematic changes in the shape of the Zn K-edge white line, which is known to be sensitive to the disorder in oxidized zinc materials.^67,68^ Moreover, in the Zn K-edge FT-EXAFS spectrum for the as-prepared pure Zn NPs we observe an additional feature at *ca.* 2.3 Å (blue vertical arrow in Fig. 4(b)) that is not pronounced in the EXAFS spectra of the as-prepared bimetallic samples. Previously this feature was attributed to an additional long Zn–O bond not present in wurtzite-type ZnO, and/or to the presence of disordered hydroxide or amorphous ZnO phases.^13,47,69^ Due to the low intensity of this feature, however, it is challenging to assign it to any particular structural motif in conventional EXAFS analysis. Results of the NN analysis of the Zn K-edge EXAFS spectra of the as-prepared samples (Fig. 5(c)) reveal that Zn-rich NPs exhibit sharper Zn–M RDF peaks, which is a signature of a more ordered structure. Moreover, an additional feature appears in the Zn–O RDF at a *R* value of *ca.* 2.8 Å (blue vertical arrow in Fig. 5(c)). Such a feature is not characteristic for wurtzite-type ZnO. Thus, in depth NN analysis reveals that even in the oxidized state, the interactions between Cu and Zn species affect the structure and properties of the catalysts.

Under CO_2_RR reaction conditions, the differences in the local structure for samples with different Cu to Zn ratios are clear in the FT-EXAFS spectra. While for monometallic Cu NPs the amplitudes of the Cu K-edge FT-EXAFS peaks are comparable to those of metallic Cu, they are reduced significantly in bimetallic samples with increasing Zn concentration. A similar trend is observed also in the Zn K-edge EXAFS data, and is consistent with the difference between the EXAFS spectra for metallic Cu, or a CuZn brass alloy with fcc-type structure, and that for metallic Zn, where the latter has a significantly lower amplitude of all EXAFS features due to a more disordered, non-close-packed structure. Note here that this disordered structure makes the interpretation of the EXAFS data in Zn-rich materials using conventional approaches especially challenging. Even for a simple metallic Zn foil, the conventional EXAFS fitting underestimates the coordination numbers by up to 50%.^13,70,71^

By our NN approach, in turn, the analysis of such disordered structures can be done easily. RDFs extracted by NN from the Cu K-edge and Zn K-edge EXAFS data (Fig. 5(b) and (d), respectively) show that both, Cu and Zn are completely reduced in the final state under CO_2_RR conditions. For the Cu-rich NPs, the RDFs obtained for Cu–M and Zn–M are similar to those in the Cu foil or the CuZn brass foil, suggesting well-ordered fcc-type structures. With increasing Zn content, a shift of all features towards larger interatomic distances is observed in both, Cu–M and Zn–M RDFs in agreement with the differences in lattice spacings in monometallic Cu and CuZn fcc-type alloys.^72^ Simultaneously, we observe a decrease in the amplitude of the 3^rd^ Cu–M and Zn–M RDF peaks, and an increase of the Cu–M and Zn–M RDF intensity at *ca.* 5 Å for Zn-rich NPs. This can be interpreted as a signature of a gradual transformation from the fcc-type structure to a disordered hcp-type structure. In fact, the Zn–Zn RDF for monometallic Zn NPs is indeed very similar to that of the Zn foil.

Using the NN approach, a very large number of experimental spectra can be processed very quickly, and we can follow the time-dependent evolution of the local structure around the catalytically active species, as they get reduced under reaction conditions (Fig. 7). In particular, for Zn species in bimetallic CuZn catalysts we observe a relatively slow reduction (within several hours), indicated by a gradual disappearance of the Zn–O contribution in the NN-yielded RDFs, and an increase in the amplitude of the Zn–M features characteristic for the metallic phase, Fig. 7(a). We can also track these changes by analysing the Zn–O and Zn–Zn coordination numbers obtained by integration of the corresponding RDF peaks, Fig. 7(b–e). Note here that Zn–O and Zn–Zn coordination numbers are obtained independently, but their trends are in a good agreement: the reduction of the Zn–O coordination number values from 4 to 0 is in all cases accompanied by an increase in the Zn–Zn coordination number from 0 to 12 that takes place on a similar time scale. When the results for different NPs are compared, an interesting observation is that monometallic Zn NPs (Fig. 7(e)) are reduced significantly faster that bimetallic NPs, Fig. 7(b–d), suggesting that interactions between Cu and Zn affect significantly the reducibility of the catalysts. The quick reduction of the pure Zn NPs also explains their stable catalytic behaviour (Fig. 2(e)), where the selectivity towards the main CO_2_ conversion products does not change after the first few minutes. The differences in the Zn environment, however, do not explain the different time-dependencies of the selectivity for the bimetallic NPs (Fig. 2(b–d)), since the reduction rates of Zn species in all bimetallic samples are similar (Fig. 7(b–d)).

The environment of Cu, in turn, exhibits quite different trends from those of Zn. First of all, Cu is reduced almost immediately in all samples, and already after the first EXAFS scan, Cu is completely metallic, with no detectable Cu–O contribution in the NN-yielded results. After this quick initial reduction, however, the structure around Cu continues to change (Fig. 6 and S5†) in the bimetallic catalysts, with the positions of all Cu–M RDF peaks shifting towards larger interatomic distances. This trend is the most pronounced for the Cu_50_Zn_50_ NP catalysts, where immediately after potential application, the positions of all RDF peaks match those in metallic Cu, while after several hours under CO_2_RR conditions, all peak shifts towards larger interatomic distances and match those in the RDF for CuZn brass, which has the same fcc-type structure as metallic Cu, but expanded lattice constants (Fig. 6(a)). The changes in the positions of the RDF peaks (Fig. 6(d)) can thus be interpreted as a signature of gradual alloying of Cu with Zn species. Immediately after the potential is applied, the Zn-species are still mostly oxidized, and the metallic phase of the catalyst consists predominantly of metallic Cu. Upon gradual reduction of Zn (Fig. 7), the metallic phase of the catalyst is enriched by Zn, forming an alloy with distinct catalytic properties. In particular, for the Cu_50_Zn_50_ NPs we observe a switch from CH_4_ to CO and then H_2_ as the main CO_2_ conversion products (Fig. 2(c)). Importantly, the changes in the catalyst structure and catalytic properties take place on the same time scale of several hours. For comparison, for monometallic Cu NPs we observe neither time-dependent changes in the catalyst structure after the initial reduction (Fig. 6(b)), nor time-dependent changes in selectivity (Fig. 2(a)), giving us additional confidence that the observed changes in the catalyst structure and selectivity are intimately linked.

Interestingly, for the Cu_70_Zn_30_ NPs, the time-dependent changes in the catalyst structure are less pronounced than for the Cu_50_Zn_50_ NPs. After the initial increase of the interatomic distances, the Cu–M bond length in the Cu_70_Zn_30_ NPs plateaus at a value in between those of metallic Cu and a CuZn brass alloy Fig. 6(c). This correlates well with the slower time-dependent selectivity changes and higher production of CH_4_, observed for this sample in Fig. 2(b), and suggests that the amount of Zn in this catalyst is not sufficient to form a Cu–Zn alloy that produces mostly mainly H_2_ and CO as observed for the Cu_50_Zn_50_ NP catalyst.

For the Cu_30_Zn_70_ NP catalyst, time-dependent structural changes are even less pronounced. After a quick initial reduction, the Cu–M distance in this sample is already close to that in CuZn brass alloy and in Cu_50_Zn_50_ NPs in their final state, Fig. 6(d and e). Consequently, also the time-dependent selectivity changes are not pronounced for this sample (Fig. 2(d)), and the distribution of reaction products is similar to those for Cu_50_Zn_50_ NP catalyst in its final state.

The change observed in the selectivity of the CuZn catalysts is in agreement with our previous work indicating that the type of interaction between the Zn and Cu component determines the products obtained.^13^ For Cu catalysts in contact with ZnO, the conversion of CO_2_ to hydrocarbons has been previously observed.^9,10^ At the same time, production of only H_2_ and CO was observed for Cu–Zn alloys.^12^ These results are in accord with the switch in the selectivity displayed here from the production of methane for Cu-rich catalysts in contact with unreduced ZnO species to the production of CO and H_2_ with increasing reaction time upon formation of the Cu–Zn alloy. When comparing our results for small (*ca.* 5 nm) CuZn NPs with literature data, particle-size effects need to be considered.^13^ The small particle sizes in our samples may explain the methane selectivity observed, in contrast to the production of C_2_ hydrocarbons shown in ref. 9 and 10. The slow restructuring of the CuZn catalysts and corresponding changes in the catalytic properties are especially pronounced for the sample with a Cu to Zn ratio close to 1. It is plausible that the zinc oxide and copper oxide species present in our as-prepared catalyst are significantly segregated, hindering and delaying the formation of a homogenous Cu–Zn alloy under reducing conditions.

## 5. Conclusions

In this work we demonstrate the potential of combining *operando* EXAFS spectroscopy and artificial neural network methods for tackling the challenging problem of monitoring the catalyst structure and composition under working conditions. We demonstrated that the features of the NN-EXAFS method such as the sensitivity to the contributions of distant coordination shells and to strongly disordered structural phases, its applicability to heterogeneous mixtures with coexisting different species, robustness to experimental noise, and applicability for quick and on-the-fly analysis can provide key answers about the structural and compositional changes in the catalyst under reaction conditions, and allow us to identify the important parameters responsible for the catalytic properties and their time-dependent changes.

In particular, in this study of time-dependent changes in CuZn nanocatalysts, we observed that the interactions between Cu and Zn affect the crystallographic structure of the as-prepared catalyst, its final state under CO_2_RR conditions (lattice spacing and crystallographic structure of the reduced catalyst), as well as the degree and rate of the time-dependent structural changes (*e.g.*, reduction rate of Zn species and the degree and rate of Cu and Zn alloying). The interplay of geometric effects (changes in the interatomic distances) and electronic effects (changes in the electronic structure due to Cu and Zn alloying) during the gradual Cu and Zn reduction and alloying, results in different catalytic selectivity and stability for Cu-rich and Cu-poor samples. We demonstrated that we can use the Cu–M interatomic distance as a convenient descriptor of the catalyst properties in these size-selected bimetallic NPs, where the NPs with shorter interatomic distance (similar to that in metallic Cu) favour the production of CH_4_, while for NPs with longer Cu–M distances the production of CO is favoured. While the Cu–M interatomic distances in the latter case are similar to those in the CuZn brass alloy, we observe signatures of deviations from fcc-type structures in the RDFs for the Zn-rich NPs, which also may contribute to the intriguing composition-dependent properties of the CuZn catalysts.

Our findings open new avenues for tuning the selectivity of bimetallic CO_2_ electroreduction catalysts *via* the rational design of their initial structure and chemical state.

## Conflicts of interest

There are no conflicts to declare.

## Supplementary Material

SC-011-D0SC00382D-s001
