# Supplementary material for: Linking the evolution of catalytic properties and structural changes in copper–zinc nanocatalysts using operando EXAFS and neural-networks
Source: Chem Sci. 2020 Mar 11;11(14):3727–36. doi: 10.1039/d0sc00382d (PMC8152410; doi:10.1039/d0sc00382d)
Supplement: SC-011-D0SC00382D-s001 [file SC-011-D0SC00382D-s001.pdf]

Electronic Supplementary Material (ESI) for Chemical Science.
This journal is © The Royal Society of Chemistry 2020

Electronic Supporting Information for

Linking the Evolution of Catalytic Properties and Structural Changes in Copper-Zinc Nanocatalysts using *Operando* EXAFS and Neural-Networks

Janis Timoshenko, Hyo Sang Jeon, Ilya Sinev, Felix Haase, Antonia Herzog and Beatriz Roldan Cuenya*

Department of Interface Science, Fritz-Haber Institute of the Max-Planck Society,

14195 Berlin, Germany

Supplementary Note 1: Details of XAS data collection

For *in-situ* X-ray absorption spectroscopy (XAS) measurements for CuZn nanocatalysts we used a home-made cell for electrochemical measurements. A platinum mesh and leak free Ag/AgCl electrode were used as counter and reference electrodes, respectively. For the XAS studies, the CuZn NPs samples were deposited on a carbon foil substrate (GoodFellow), while the other side of the substrate was covered with Kapton. The sample was mounted in the electrochemical cell

with its Kapton-covered side acting as a window, while the side coated with the CuZn catalyst was in contact with the electrolyte.

In-situ XAS measurements for $Cu_{50}Zn_{50}$ and $Cu_{30}Zn_{70}$ nanoparticles (NPs) were performed at the SAMBA beamline at SOLEIL synchrotron. XAS data at Cu K-edge (E_0 = 8979 eV) and Zn K-edge (E_0 = 9659 eV) were collected, using a Si(220) monochromator for energy selection. Higher harmonics were rejected using a Pd-coated mirror. The beam size was 1 x 0.5 mm. Measurements were performed in fluorescence mode using a 13-channel Ge detector. The intensity of the incident radiation was measured by an ionization chamber filled with a 500 mbar N_2/500 mbar He mixture. Two additional ionization chambers filled with 1700 mbar N_2 (in I_1 chamber) and 150 mbar Ar/850 mbar N_2 (in I_2 chamber) were used for measurements in transmission mode for reference samples.

In-situ XAS measurements for Cu_{100}, Zn_{100} and $Cu_{70}Zn_{30}$ NPs were performed at the CLAESS beamline at the ALBA synchrotron in a similar manner using Si(111) monochormator and 6-chanel Si drift detector, and beam size of 0.3 x 0.3 mm.

Supplementary Note 2: Details of neural network architecture and training

For neural network (NN)-based interpretation of EXAFS data in partially oxidized NPs, we use a similar procedure as previously developed for mono- and bimetallic compounds.[1, 2] To generate and train the NN we rely on the off-the-shelf available NN implementation, available within Wolfram Mathematica 11.3.[3] For NN training we use theoretical Cu K-edge EXAFS spectra, obtained in classical molecular dynamics (MD) simulations (see **Supplementary Note 3**).

The so called hyperparameters of the NN (e.g., number of NN layers and number of nodes in each layer) are obtained by a manual grid search, with the goal to optimize the performance of the

NN on a validation set, i.e., a set of spectra for which the corresponding true structure is known, but that was not used for NN training.

Our NN consists of an input layer, five hidden layers with 450 nodes in each, and an output layer. A hyperbolic tangent function is used as activation function. The batch size for NN training is 512. We perform training for 300 rounds. The loss function is defined as L_2-norm between NN output and the true radial distribution function (RDF) value, averaged over all examples in the batch. The "ADAM" method (stochastic gradient descent with adaptive with adaptive learning rate) was employed with default parameters β_1=0.9 and β_2 = 0.999, is used for the optimization of NN weights.

Similarly as in our previous works,[1, 2] the nodes in the input layer are set to the values of experimental or theoretical EXAFS spectra, which are mapped via Morlet wavelet transform[4, 5] (WT) to (k,R) space. The integration of spectra for WT is carried out in the range between k_{min} = 3 $Å^{-1}$ and k_{nax} = 11 $Å^{-1}$. To reduce the influence of systematic errors and also to reduce the NN size, for each point in the (k,R) space we compare the corresponding absolute WT values for a subset of training spectra, and use as inputs for the NN only those ca. 800 points that have variance larger than 10% of the maximal observed variance. Each WT point is encoded by two NN input nodes, containing the real and imaginary parts of complex Morlet WT value, resulting in ca. 1600 nodes in the NN input layer.

The output nodes of the NN correspond to the concatenated lists of bin heights for histograms of Cu—O and Cu—Cu bond lengths, calculated in the range between R_{min} = 1.0 Å and R_{max} = 5.5 Å and with bin size ΔR. The latter determines the resolution of our method in R-space and is limited by the k-range used for analysis to $1/(2k_{max} - 2k_{min})$.[6] Therefore, here we use ΔR=0.06 Å, which results in $2 \cdot 76 = 152$ output nodes.

To estimate the uncertainties of the NN-yielded RDFs, we train independently several NNs using different subsets of training sets, and compare their predictions. The average value is reported as the final NN-yielded answer, while the standard deviation of NNs predictions is used to calculate the error bars.

The NN training was carried out on a personal computer and took ca. 30 min per NN.

Supplementary Note 3: Molecular dynamics simulations and generation of theoretical EXAFS spectra for NN training

For NN training we use theoretical EXAFS spectra, obtained in classical MD simulations. MD is carried out using the GULP code.[7, 8] See Ref.[9] for technical details on the MD simulations and EXAFS calculations. MD-EXAFS simulations were carried out at LASC cluster.[10] Several CPU hours were needed per each MD simulation.

To define forces acting within Cu—Cu, Cu—O and O—O atomic pairs, we tried out the Sutton-Chen (SC) force field model,[11] Lennard-Jones (LJ) type potential[12] and reaxFF-type potentials.[13] Default values for parameters for SC and reaxFF-type potentials were used, as provided within GULP code. Parameters for LJ-type potential were taken from Ref.,[12] but with ε_0 parameter for O—O bond increased up to 0.2754 eV, since the structures obtained with the original ε_0 value were not stable. Examples of EXAFS spectra for bulk reference materials, calculated with these interaction models, together with the corresponding RDFs are shown in **Figure S1** and **Figure 1** in the main text.

When the calculated EXAFS spectra are compared with experimental data, **Figure 1(a)**, one can notice that the agreement for Cu_2O and CuO oxide materials is not as good as that obtained for metallic systems employing the SC potential. Moreover, we have found that the currently widely

used reaxFF potential[13] fails to represent the dynamics and structure of Cu_2O as probed by EXAFS, and is outperformed in this case by a simple LJ-type potential, which is therefore used for the Cu_2O models in **Figures 1(a)** and **S1**. Somewhat better agreement between the reaxFF-based model and experimental Cu K-edge EXAFS was obtained for CuO and, especially, for copper hydroxide $Cu(OH)_2$.

Despite the noticeable disagreement between theoretical and experimental EXAFS, we have found that Cu—O and Cu—Cu RDFs obtained in these MD simulations, are in fact quite reasonable. To demonstrate that, in **Figure 1(b,c)** in the main text we compare RDFs for MD models with the ones independently extracted from experimental EXAFS spectra by a reverse Monte Carlo approach (RMC). [9, 14-16] RMC is an iterative stochastic structure fitting method, which relies on random modifications of the initial atomistic structure model until a good agreement between the calculated structure-sensitive data (EXAFS spectra in our case) and the experimental measurements is obtained. Note here that RMC can only be used for the interpretation of EXAFS spectra in relatively simple, well-defined homogeneous materials, because the initial structure model needs to be specified. RMC is not suitable for studies of materials experiencing structural transformations, and it is also computationally expensive, requiring several CPU days of calculations for the interpretation of a single spectrum. RMC is useful here, however, for validation of the accuracies of the MD and NN methods. RMC-EXAFS simulations are carried out using EVAX code.[14] See Ref.[17] for the technical details of RMC simulations.

As shown in **Figure 1(a)** in the main text, 3D structure models, extracted from RMC analysis, provide much better match with experimental EXAFS data than MD simulations. The RDFs, extracted from RMC models, however, are in a good agreement with those from MD. This allows us to conclude that the differences between MD-EXAFS and experimental EXAFS for oxide

materials in **Figure 1(a)** are mostly due to many-atomic contributions to EXAFS data (multiple-scattering effects), which are not represented by RDFs, and/or minor inaccuracies in the atomic thermal motion amplitudes in the MD models. For example, the noticeably lower amplitude of the first peak in the Fourier-transformed MD-EXAFS for CuO, **Figure 1(a)**, is a result of systematically underestimated Cu—O bond strength in the reaxFF model, which is responsible also for the broader first peak in the Cu—O RDF than that obtained from the RMC-based analysis of the corresponding experimental EXAFS data, **Figure 1(b)**.

Nevertheless, the overall resemblance between RDFs from MD simulations and those extracted from experimental data for the reference oxide materials allows us to use the MD simulations to generate training spectra for our NN method. Let us emphasize here that in our approach we need only realistic-looking theoretical EXAFS data, and do not require a perfect agreement between simulated and experimental data. Theoretical data are used only to train a NN and to establish the relationship between spectral features and materials structure, and are not used to directly match the results of experimental measurements. However, the accuracy of the NN predictions needs to be carefully validated by applying it to the analysis of experimental data for reference materials, for which the corresponding structure is known, as we do below (**Supplementary Note 4**).

As in our previous works,[2, 18, 19] we have also carried out MD simulations at different temperatures to introduce different degree of disorder in our models, and have rescaled the atomic coordinates in the MD models in the range between 80% and 120% to account for possible differences in lattice spacings in our MD models and experimentally investigated samples, ending up with ca. 12000 unique structure models and corresponding EXAFS spectra. We do not use them directly for the NN training, but instead combine them in random linear combinations, employing the fact that for a mixture of several species with concentrations w_i, both corresponding EXAFS

spectra $\chi(k)$ and partial RDFs $g_p(R)$ can be expressed as $\chi(k) = \sum_i w_i \chi_i(k)$ and $g_p(R) = \sum_i w_i g_{p,i}(R)$, where $\chi_i(k)$ and $g_{p,i}(R)$ are spectra and RDFs for pure compounds, respectively. The use of linear combinations allows us to further expand the training set and generate an almost unlimited number of training examples (60000 such examples are used for NN training in this case), and allows our method to work with EXAFS spectra of heterogeneous samples and mixtures of different phases.

Finally, to account for the fact that photoelectron reference energy E_0 value (see **Eq.(1)** in the main text) can change from sample to sample due to, e.g., reduction/oxidation of the sample components, each of the training EXAFS spectra is artificially shifted by a random ΔE_0 value in the range between +10 and -10 eV. Thus we ensure that the NN does not assign any physical meaning to a small shift of the energy scale in the experimental EXAFS data. We have demonstrated before[20] that such a simple approach allows one to address the uncertainty of the E_0 values in experimental data.

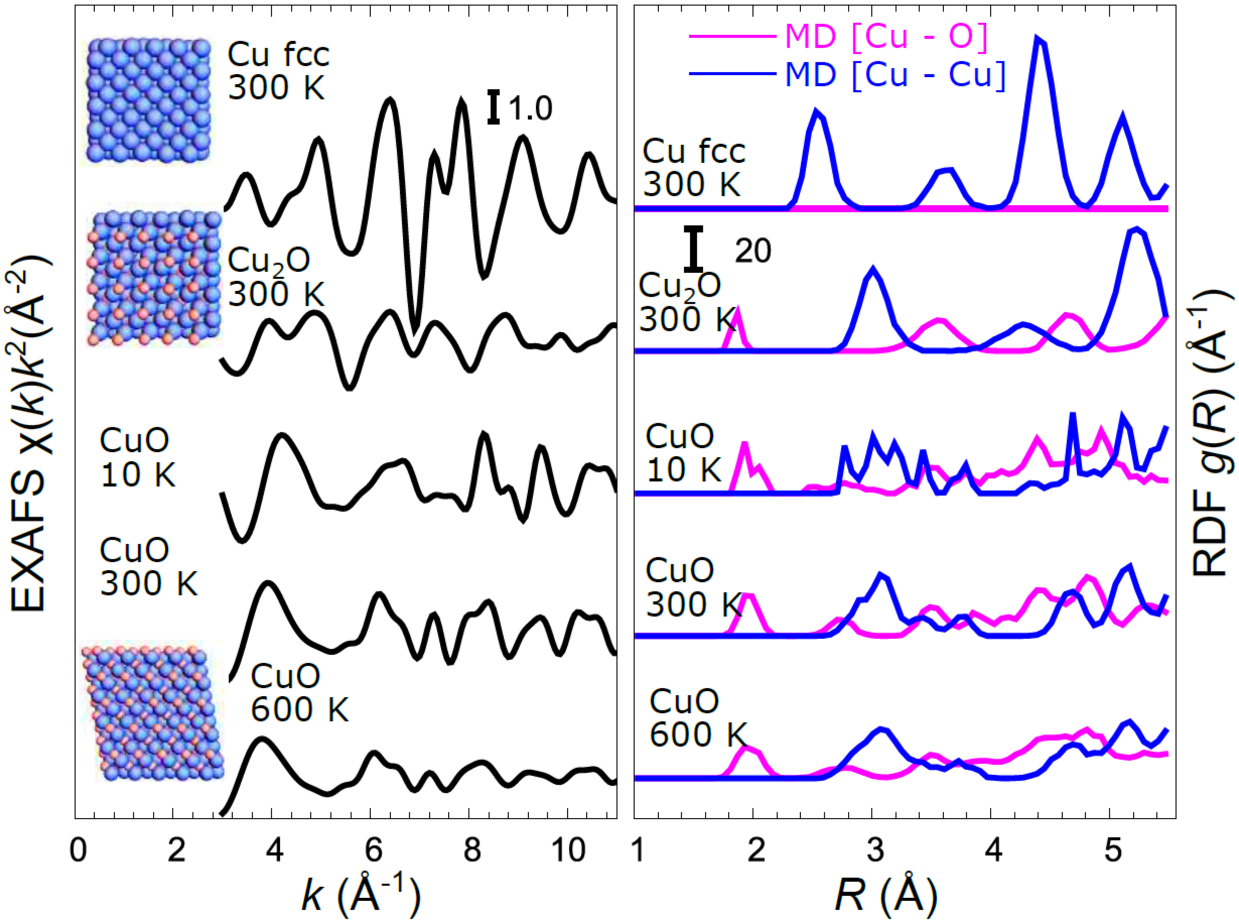

Figure S1. Examples of EXAFS spectra used for the NN training: theoretical Cu K-edge EXAFS data, calculated in MD simulations for metallic Cu (Sutton-Chen type potential), Cu_2O (LJ-type potential) and CuO (reaxFF-type potential) (a) and corresponding partial Cu-O and Cu-Cu RDFs (b). Insets show snapshots of the corresponding MD models (Cu atoms – large blue spheres, O atoms – small red spheres). The simulations are carried out at different temperatures to introduce different degrees of disorder in the model. Spectra and RDFs are shifted vertically for clarity.

Supplementary Note 4: Validation of NN-EXAFS method with RMC data

To validate the accuracy of our NN method, in **Figure S2** we apply it for the interpretation of experimental EXAFS data for metallic and non-metallic reference compounds: metallic Cu at different temperatures, Cu_2O and CuO oxides, $Cu(OH)_2$ hydroxide. The RDFs, yielded by NN, are compared with the results of RMC simulations, also carried out for these materials. The average predictions of three independently trained NNs are reported as thick solid lines in **Figure S2** (and other figures here and in the main text), while the uncertainty (standard deviation of NNs predictions) is reflected by shaded region in **Figure S2**. Good agreement (within uncertainty) between RMC and NN results for such different compounds as shown in **Figure S2**, gives us confidence in the accuracy of our NN method for a broad range of materials with different degree of disorder. Moreover, while our NN is trained on MD-EXAFS data for Cu and copper oxides only, due to the similarity of the scattering functions of elements that are neighbors in the Periodic Table, it can be used to extract Cu-N contribution in copper nitride Cu_3N (**Figure S2(b)**) and total Cu-metal (Cu-M) contribution in copper-zinc brass alloy (**Figure S2(a)**). Even more so, our NN, trained on Cu K-edge EXAFS data, can be immediately applied for the interpretation of EXAFS data of Zn and Ni (that are neighbors of Cu in Periodic Table), and gives reliable results, when used for interpretation of EXAFS data in metallic Ni, Zn, Zn-Cu alloys, as well as zinc and nickel oxides, **Figures S2(a),(c)**.

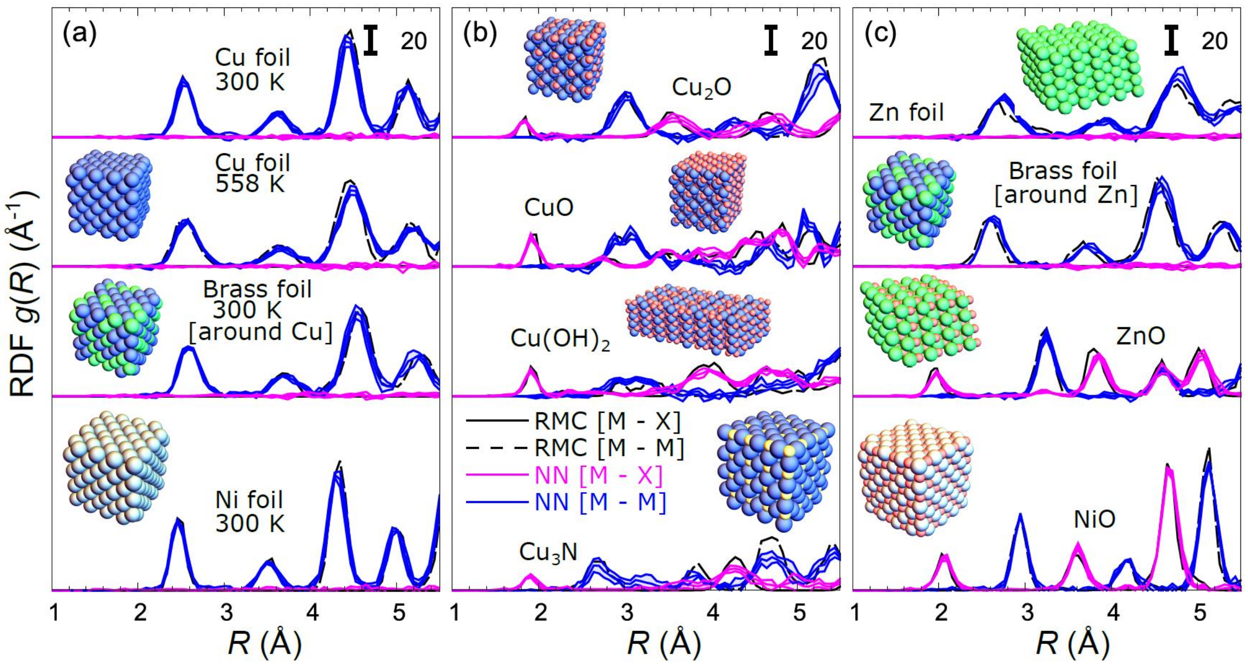

Figure S2. Validation of the NN accuracy with RMC data. Cu-X (X = O or N) and Cu-M (M = Cu, Zn or Ni) partial RDFs, extracted by a NN from experimental Cu K-edge (a,b), Ni K-edge (a, c) and Zn K-edge (c) EXAFS data. Insets show the final structure models, obtained in RMC calculations. RDFs are shifted vertically for clarity.

To demonstrate the accuracy of the former approach not just for pure compounds, but also for their heterogeneous mixtures, in **Figure S3** we use a NN to extract Cu—O and Cu—Cu RDFs from model spectra, constructed as linear combinations of experimental Cu K-edge spectra for a Cu foil and CuO with weights w_{Cu} and (1-w_{Cu}), correspondingly, **Figure S3(a)**. By varying the weight of the metallic contribution, w_{Cu}, we mimic EXAFS spectra for mixtures of coexisting reduced and non-reduced species. In **Figure S3(b)**, the RDFs yielded by the NN are compared with the corresponding properly weighted linear combinations of RDFs from RMC simulations for pure Cu and CuO compounds (see **Figure S2(b)**). For a more quantitative comparison we estimate average Cu—O and Cu—Cu coordination numbers by integrating the first peak in Cu—O and Cu—Cu RDFs. Integration limits are indicated by vertical dashed lines in **Figure S3(b)**.

The obtained coordination numbers and their dependency on w_{Cu} are shown in **Figures S3(c)** and **(d)**. The expected true dependency is shown by solid black lines, which reflect the linear change in the Cu—O coordination number from 4 (in pure CuO) to 0 (in pure metallic Cu), and that in the Cu—Cu coordination number from 0 (in pure CuO) to 12 (in pure fcc-type metallic Cu). Good agreement between these linear dependencies and the NN results demonstrates the accuracy of our NN and its applicability for quantitative analysis of heterogeneous mixtures.

After NN validation is completed, we can apply the method for the interpretation of real experimental data for bimetallic catalysts. Experimental Cu K-edge and Zn K-edge EXAFS data for CuZn nanocatalysts are shown in **Figure S4** in the main text. The results (Cu-O and Cu-M RDFs, where M = Cu or Zn), obtained from Cu K-edge EXAFS data are shown in **Figure S5** and **Figures 5(a,b)** and **6(a)** in the main text. Changes in the position of the 1st peak in Cu-M RDF, corresponding to the increase of the Cu-M interatomic distance due to gradual Cu and Zn alloying, are shown in **Figure S6** and **Table S1**. The corresponding changes in the position of the 3rd peak in the Cu-M RDF are also given in **Table S1**, and plotted in **Figure 6** within the main text. Zn-O and Zn-M RDFs, extracted from Zn K-edge EXAFS data, are shown in **Figure S7** and in **Figures 5(c,d)** and **7(a)** in the main text. The time-dependencies of the integrated areas under the 1st Zn—O RDF peak (1st shell Zn—O coordination number) and under the 1st Zn—M RDF peak (1st shell Zn—M coordination number) for bimetallic CuZn nanocatalysts and for the monometallic Zn_{100} sample are shown in **Table S2** and in **Figure 7(b-e)** of the main text. See the main text for detailed discussion of the obtained results.

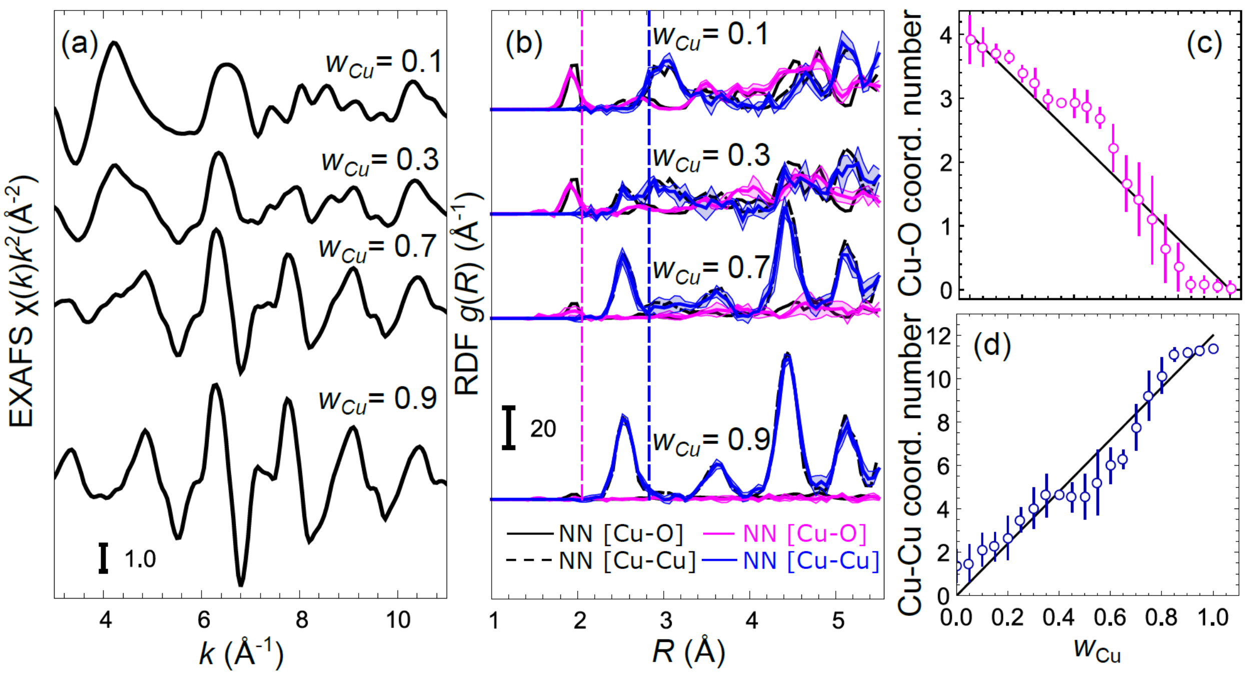

Figure S3. Validation of the NN accuracy for heterogeneous mixtures. Model EXAFS spectra obtained by linearly combining experimental Cu K-edge EXAFS of a Cu foil and CuO (b). Cu—O and Cu—Cu RDFs yielded by NN for the spectra shown in (a) are compared with linear combinations of the corresponding RDFs extracted by RMC from experimental EXAFS data for pure compounds (b). First shell Cu—O (c) and Cu—Cu (d) coordination numbers are obtained by integrating RDFs shown in (b) up to the *R* value, indicated by vertical dashed lines in (b).

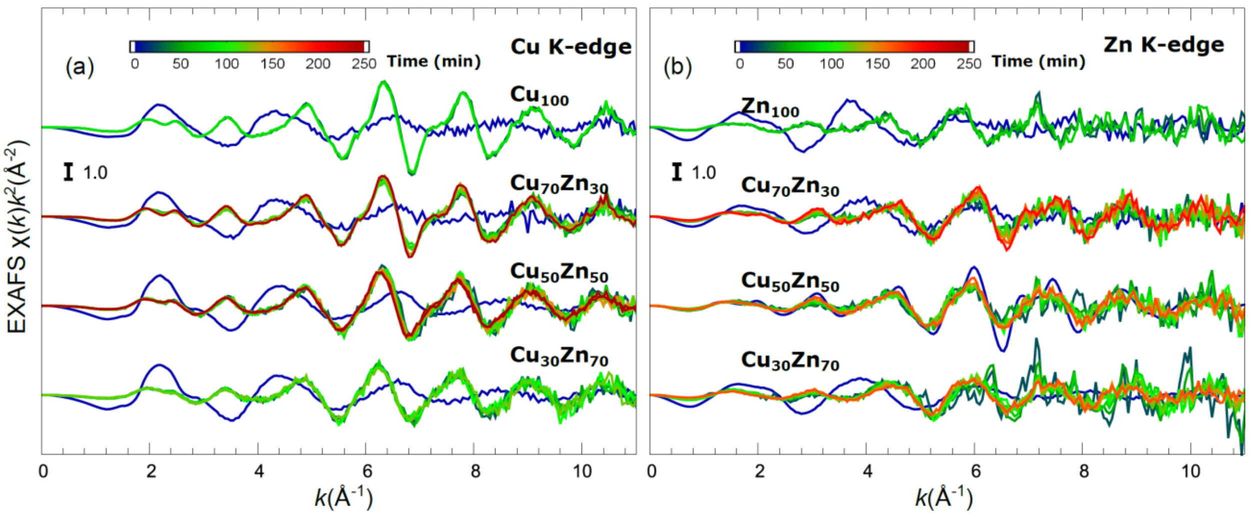

Figure S4. Time-dependent Cu K-edge (a) and Zn K-edge (b) EXAFS spectra for Cu_{100}, Zn_{100}, $Cu_{70}Zn_{30}$, $Cu_{50}Zn_{50}$ and $Cu_{30}Zn_{70}$NPs. Spectra are shifted vertically for clarity.

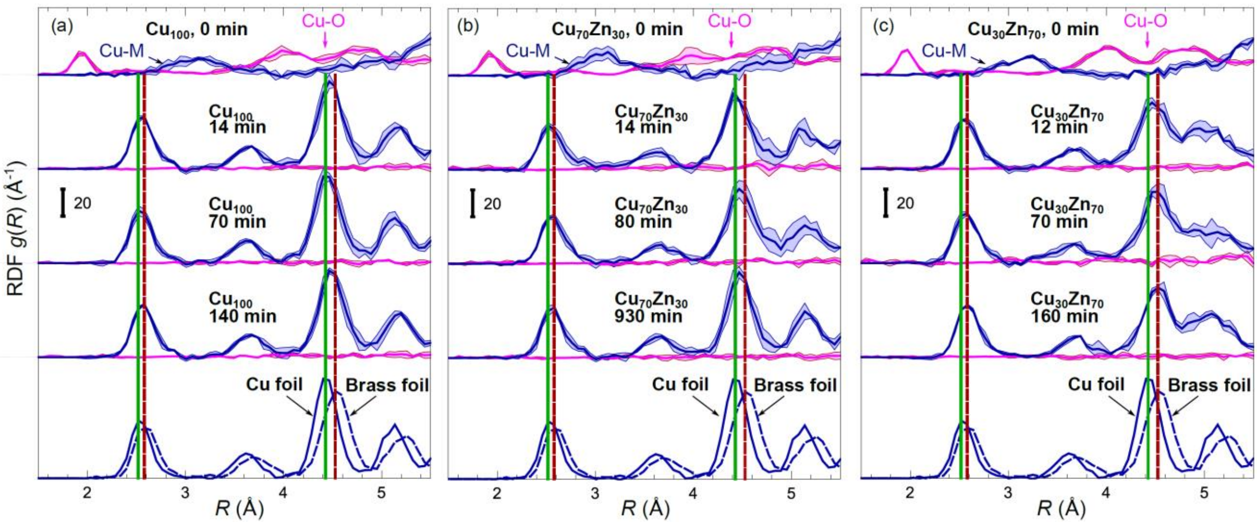

Figure S5. Evolution of the partial RDFs for the Cu_{100} NP sample (a), $Cu_{70}Zn_{30}$ NPs (b) and $Cu_{30}Zn_{70}$ NPs (c), as extracted by NN from time-dependent Cu K-edge EXAFS data (see Figure 4(a) in the main text and Figure S4(a)). RDFs extracted from the Cu K-edge EXAFS for Cu foil and CuZn brass foil are shown for comparison. Vertical solid and dashed lines show the positions of maxima for the 1st and 3rd RDF peaks in Cu foil and CuZn brass foil, respectively.

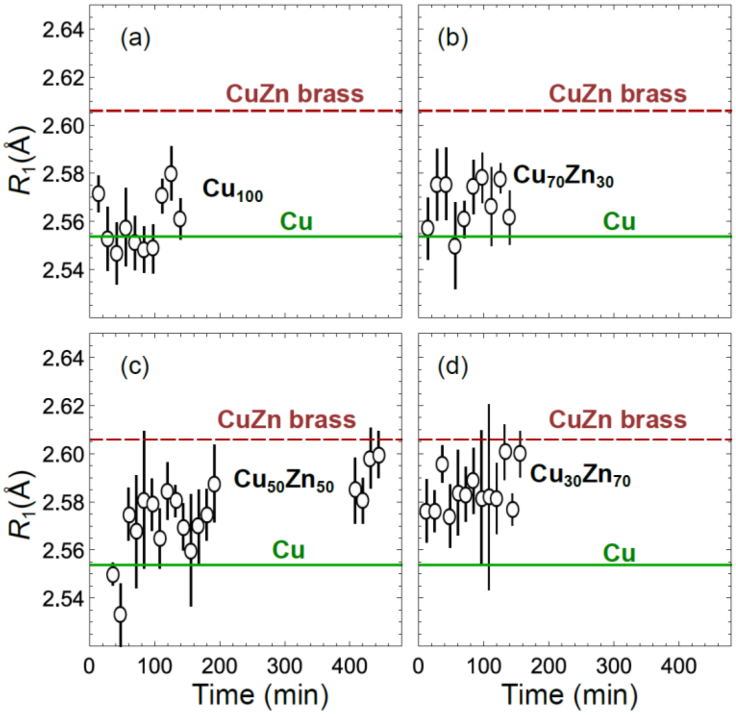

Figure S6. Time-dependencies of the positions of the 1st peak in Cu—M RDF for Cu_{100} NPs (a), $Cu_{70}Zn_{30}$ NPs (b), $Cu_{50}Zn_{50}$ NPs (c) and $Cu_{30}Zn_{70}$ NPs (d), as extracted by NN from time-dependent Cu K-edge EXAFS data (see Figure 4(a) in the main text and Figure S4(a)).

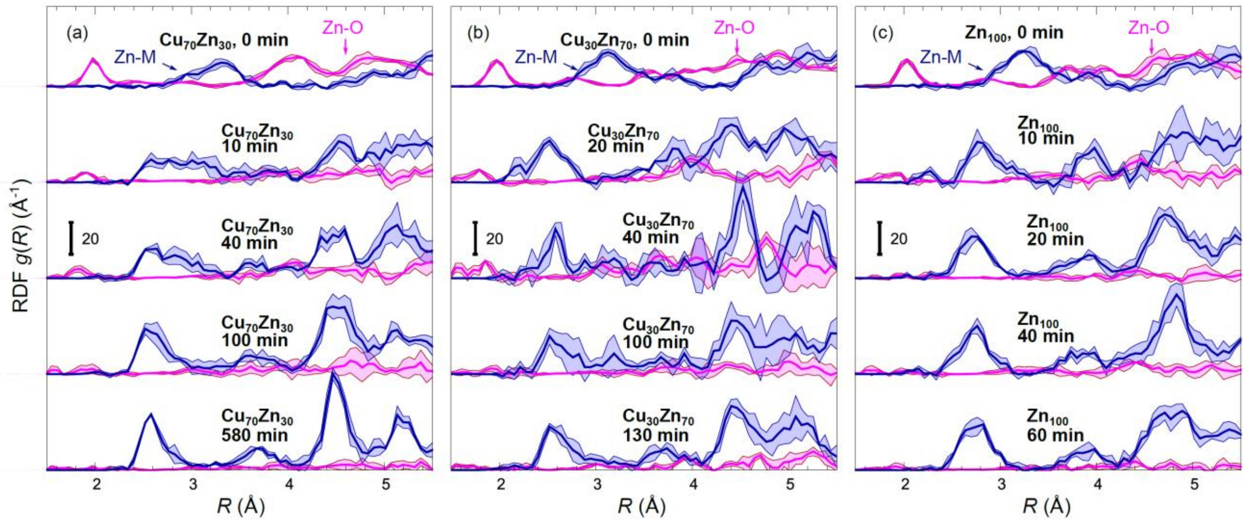

Figure S7. Evolution of the partial RDFs for $Cu_{70}Zn_{30}$ NPs (a), $Cu_{30}Zn_{70}$ NPs (b) and Zn_{100} NPs (c), as extracted by NN from time-dependent Zn K-edge EXAFS data (see Figure 5(a) in the main text and Figure S5(a)).

Table S1. Time-dependencies of the positions of the 1st peak (R_1) and 3rd peak (R_3) in Cu—M RDF for Cu_{100} NPs (a), $Cu_{70}Zn_{30}$ NPs (b), $Cu_{50}Zn_{50}$ NPs (c) and $Cu_{30}Zn_{70}$ NPs (d), as extracted by NN from time-dependent Cu K-edge EXAFS data (see Figure 4(a) in the main text and Figure S4(a)).

Cu_{100}			$Cu_{70}Zn_{30}$			$Cu_{50}Zn_{50}$			$Cu_{30}Zn_{70}$		
Time (min)	R_1(Å)	R_3(Å)	Time (min)	R_1(Å)	R_3(Å)	Time (min)	R_1(Å)	R_3(Å)	Time (min)	R_1(Å)	R_3(Å)
14	2.57(1)	4.46(2)	14	2.56(1)	4.43(2)	36	2.55(1)	4.42(1)	12	2.58(1)	4.48(2)
28	2.55(1)	4.43(2)	28	2.58(1)	4.47(2)	48	2.53(1)	4.40(2)	24	2.58(1)	4.49(2)
42	2.55(1)	4.42(2)	42	2.58(1)	4.46(3)	60	2.57(1)	4.48(3)	36	2.60(1)	4.50(2)
56	2.56(2)	4.43(3)	56	2.55(2)	4.43(4)	72	2.57(2)	4.45(3)	48	2.57(1)	4.48(3)
70	2.55(1)	4.42(2)	70	2.56(1)	4.46(2)	84	2.58(3)	4.48(4)	60	2.58(2)	4.49(2)
84	2.55(1)	4.42(2)	84	2.57(1)	4.47(3)	96	2.58(1)	4.47(3)	72	2.58(1)	4.51(2)
98	2.55(1)	4.42(2)	98	2.58(1)	4.47(2)	108	2.56(1)	4.46(2)	84	2.59(1)	4.50(2)
112	2.57(1)	4.46(2)	112	2.57(2)	4.45(2)	120	2.58(1)	4.49(2)	96	2.58(3)	4.49(5)
126	2.58(1)	4.48(2)	126	2.58(1)	4.47(1)	132	2.58(1)	4.48(2)	108	2.58(4)	4.48(5)
140	2.56(1)	4.44(1)	140	2.56(1)	4.45(1)	144	2.57(1)	4.47(1)	120	2.58(1)	4.49(2)
			874	2.58(1)	4.47(2)	156	2.56(2)	4.44(4)	132	2.60(1)	4.53(3)
			888	2.58(1)	4.47(2)	168	2.57(1)	4.46(2)	144	2.58(1)	4.48(2)
			916	2.58(1)	4.47(2)	180	2.57(1)	4.47(2)	156	2.60(1)	4.51(1)
			930	2.57(2)	4.46(3)	192	2.59(2)	4.50(2)			
						408	2.58(1)	4.49(2)			
						420	2.58(1)	4.49(1)			
						432	2.60(1)	4.51(2)			
						444	2.60(1)	4.51(1)			

Table S2. The time-dependencies of the integrated areas under the 1st Zn—O RDF peak (1st shell Zn—O coordination number) and under the 1st Zn—M RDF peak (1st shell Zn—M coordination number) for bimetallic CuZn nanocatalysts and for monometallic Zn_{100} NP sample, as extracted by NN from time-dependent Zn K-edge EXAFS data (see Figure 5(a) in the main text and Figure S5(a)).

$Cu_{70}Zn_{30}$			$Cu_{50}Zn_{50}$			$Cu_{30}Zn_{70}$			Zn_{100}		
Time (min)	Zn-O	Zn-M	Time (min)	Zn-O	Zn-M	Time (min)	Zn-O	Zn-M	Time (min)	Zn-O	Zn-M
0	3.1(2)	1.1(3)	0	1.9(2)	0.4(2)	0	3.4(1)	2.0(6)	0	2.3(3)	1.4(4)
8	3.8(4)	1.1(3)	8	2.8(3)	2.4(2)	8	3.4(1)	1.7(8)	11	0.9(2)	10.9(5)
16	2.7(5)	6.3(1)	16	1.8(4)	5.4(6)	16	3.4(1)	1.5(6)	22	0.2(1)	11.4(2)
24	1.6(4)	6.5(9)	24	2.2(1)	4.4(3)	24	3.5(2)	1.8(4)	33	0.4(2)	11.2(7)
32	1.0(4)	7.0(7)	32	1.9(3)	4.8(5)	32	3.6(3)	1.3(4)	44	0.2(1)	10.6(1)
40	1.0(3)	7.4(5)	40	0.0(3)	10.6(5)	40	2.3(4)	4.6(8)			
48	1.6(4)	5.1(1)	48	2.0(3)	6.3(8)	48	0.2(2)	11.1(2)			
56	0.8(3)	8.6(1)	56	0.8(0)	8.7(3)	56	0.4(1)	10.6(3)			
64	1.9(2)	6.0(1)	64	0.9(5)	7.6(3)	64	0.6(2)	9.4(8)			
72	1.2(6)	7.0(9)	72	1.4(4)	6.9(2)	72	0.4(2)	10.8(1)			
80	0.6(2)	8.5(9)	80	0.8(3)	7.7(2)	80	0.2(1)	11.1(6)			
88	1.9(3)	4.3(4)	88	0.2(1)	10.5(9)	88	0.3(2)	10.8(5)			
96	0.9(4)	9.3(1)	96	0.3(2)	10.8(4)	96	0.4(1)	10.6(1)			
104	0.2(1)	10.8(3)	104	0.4(2)	9.6(1)	536	0.3(2)	10.9(2)			
112	0.4(2)	11.2(9)	112	0.3(2)	10.6(7)	544	0.2(1)	11.3(4)			
120	0.2(1)	11.2(8)	120	0.2(2)	11.5(5)	552	0.2(1)	11.2(4)			
128	0.1(1)	11.(5)	128	0.2(1)	10.8(3)	560	0.1(1)	11.2(3)			
			136	0.1(1)	11.3(5)	568	0.1(1)	11.3(2)			
			144	0.1(1)	11.2(3)	576	0.1(1)	11.4(1)			
						584	0.1(1)	11.1(2)			

REFERENCES

1. J. Timoshenko, A. Anspoks, A. Cintins, A. Kuzmin, J. Purans and A. I. Frenkel, *Phys. Rev. Lett.*, 2018, **120**, 225502.
2. J. Timoshenko, C. J. Wrasman, M. Luneau, T. Shirman, M. Cargnello, S. R. Bare, J. Aizenberg, C. M. Friend and A. I. Frenkel, *Nano Lett.*, 2019, **19**, 520-529.
3. *Wolfram Mathematica 11.3*, 2018, Wolfram Research Inc., Champaign, Illinois.
4. H. Funke, A. Scheinost and M. Chukalina, *Phys. Rev. B*, 2005, **71**, 094110.
5. J. Timoshenko and A. Kuzmin, *Comp. Phys. Commun.*, 2009, **180**, 920-925.
6. E. A. Stern, Y. Ma, O. Hanske-Petitpierre and C. E. Bouldin, *Phys. Rev. B*, 1992, **46**, 687-694.
7. J. D. Gale, *Journal of the Chemical Society, Faraday Transactions*, 1997, **93**, 629-637.
8. J. D. Gale and A. L. Rohl, *Molecular Simulation*, 2003, **29**, 291-341.
9. J. Timoshenko, K. R. Keller and A. I. Frenkel, *J. Chem. Phys.*, 2017, **146**, 114201.
10. A. Kuzmin, *Computer Modelling and New Technologies*, 2003, **7**, 7-15.
11. A. Sutton and J. Chen, *Phyl. Mag. Lett.*, 1990, **61**, 139-146.
12. A. Hallil, J.-M. Raulot and M. Cherkaoui, *Comp. Mat. Sci.*, 2014, **81**, 366-373.
13. A. C. Van Duin, V. S. Bryantsev, M. S. Diallo, W. A. Goddard, O. Rahaman, D. J. Doren, D. Raymand and K. Hermansson, *J. Phys. Chem. A*, 2010, **114**, 9507-9514.
14. J. Timoshenko, A. Kuzmin and J. Purans, *J. Phys.: Condens. Matter*, 2014, **26**, 055401.
15. J. Timoshenko, Z. Duan, G. Henkelman, R. Crooks and A. Frenkel, *Annu. Rev. Anal. Chem.*, 2019, **12**, 501-522
16. S. Gurman and R. McGreevy, *J. Phys.: Condens. Matt.*, 1990, **2**, 9463.
17. J. Timoshenko and A. I. Frenkel, *Catal. Today*, 2017, **280**, 274-282.
18. J. Timoshenko, D. Lu, Y. Lin and A. I. Frenkel, *J. Phys. Chem. Lett.*, 2017, **8**, 5091-5098.
19. J. Timoshenko and A. I. Frenkel, *ACS Catal.*, 2019, **9**, 10192-10211.
20. J. Timoshenko, M. Ahmadi and B. Roldan Cuenya, *J. Phys. Chem.C*, 2019, **123**, 20594-20604.
